# The Institutional Library

**Published:** 1918-04-06

**Authors:** 


### THE INSTITUTIONAL LIBRARY.

**Three Clinical Studies in Tuberculous Predisposition.** By W. C. Rivers, M.R.C.S., D.P.H. (London: George Allen and Unwin, Ltd. 1917. Pp. 272. Price 12s. 6d. net.)

In three clinical studies Dr. W. C. Rivers has set out to prove by an elaborate statistical investigation that certain conditions are signs of a predisposition to consumption, and are, in fact, part of a tuberculous diathesis; as he says, "the main conclusion" of his work "can be expressed in three words—Back to diathesis."

The first study deals with the relationship of ichthyosis to consumption. The author found ichthyosis in 1.4 per cent. of 1,600 consumptives, and in .2 per cent. out of 500 controls from a similar age and social class; he comes to the conclusion that the skin affection is not caused by the tuberculosis, but that ichthyosis is a stigma of degeneracy, and that degenerates are especially liable to consumption. As ichthyosis is strongly hereditary, it follows that "the predisposition to tubercle which sometimes accompanies it may possibly sometimes be hereditary too"; and he includes a full series of genealogical tables showing the inheritance of the two diseases.

Similarly, in the second study, Dr. Rivers shows that among his cases squint was about three times as frequent in consumptives as in the general population; as any causal connection seems impossible here, he concludes that strabismus also is a stigma of degeneracy, and discusses the relationship between it and left-handedness and idiocy.

The third study is of much greater importance, for it deals with the relationship between consumption and nasal abnormality, and occupies the larger part of the book. It is *a priori* probable that loss of the filtering function of the nose should favour the inhalation of tubercle bacilli either directly into the lungs or on to the absorbent surfaces of the fauces and nasopharynx; the matter has been much in the minds of rhinologists, and an exhaustive inquiry into it cannot fail to be of value. The statistics deal with 500 sputum-positive consumptives and 452 non-tuberculous controls; intranasal abnormality occurred in 68 per cent. of the former, against 36 per cent. of the latter class, and was slightly more common in females. The material was then divided into four groups: (1) characterised by nasal obstruction or mouth-breathing; (2) by bilateral nasal atrophy; (3) other rhino-pathological cases; (4) those with normal nasal condition. The first group gave the following percentages of nasal obstruction or mouth-breathing: Consumptives, men 44 per cent., women 32 per cent.; control, men 25 per cent., women 12 per cent.; and is both more frequent in consumptives and generally commoner in men. In the second group, of intranasal atrophy, the sex-incidence is reversed: Consumptives, men 8 per cent., women 30 per cent.; control, men 1 per cent., women 12 per cent.

The author includes as an abnormality under the latter designation all cases in which the posterior wall of the nasopharynx is visible from the nostril; though many rhinologists will not agree that this condition is necessarily pathological, still the criterion is the same in the two classes, and these figures show its relative frequency in consumptives. For true atrophic rhinitis with crusting and fœtor the numbers are too small to be of much value, four in 500 consumptives, one in 452 controls; it is pretty generally believed that this affection predisposes to consumption, but Dr. Rivers does not agree with those who consider that it is a manifestation of tuberculosis.

The last two groups do not call for analysis here, but there is an interesting account of septic disease of the apex of the lung, which imitates phthisis and is caused by inhalation of pus from the nasal accessory sinuses.

The author's practice is to operate for nasal obstruction, particularly for septal deviation, in his consumptive patients; but, if the tuberculosis is beyond the Turban-Gerhardt Stadium I., quiescence is a pre-requisite, and is judged by the temperature morning, evening, and after moderate exercise. He has seen no bad results follow 100 submucous resections under local anæsthesia. He finds that nasal abnormality is not more common in patients with laryngeal tuberculosis than among other consumptives.

Some of the conclusions may be considered too sweeping. Thus, the parents of a child with ichthyosis or squint should be told to send it to an open-air school, and to bring it up to outdoor employment; all ichthyotics should be rejected at once for Army, Navy, bank, post-office, etc.; women with nasal obstruction should be penalised in life insurance, and should not be engaged for public indoor employments until the obstruction has been remedied. The author even considers these predisposing causes of value in diagnosis, and mentions the case of a girl with very indefinite signs, but with nasal obstruction, and "as nasal obstruction is so infrequent in non-tuberculous women she was at once recommended for the sanatorium."

The book ends with a table, occupying fifty-seven pages, giving details of the 500 consumptives, and is very long and exhaustive, perhaps almost too much so, but it is very carefully written, and will give the physician and the rhinologist much food for thought.

**Memoranda on Army General Hospital Administration.** By P. Mitchell. (London: Baillière, Tindall and Cox. 5s. net.)

This little book is most excellently presented, and teems with sound practical suggestions. The section that deals with the surgical division is the fullest of detail, and the best. It contains excellent lists of instruments. There are many suggestions for economies. All the suggestions are evidently based on experience. The administrator of any hospital, civil or military, will find himself in profit from a few hours' perusal of this book. A good deal of stress is laid on the desirability of giving major rank to specialists in clinical subjects of all kinds. The reason for giving the rank seems to be to assure the specialists a high rate of pay. None will dispute that a specialist should be well paid, but Army rank is given for fitness to command, in theory at any rate. It does not at all follow that a skilled anæsthetist is an able manager of men, capable of giving orders clearly, and insisting on those orders being strictly carried out. But it is the senior officer who has to enforce orders. So the anæsthetist might find himself in the position of having to enforce discipline under regulations of which he had no experience, and over the heads of Regular captains who could do it better far than he. Surely it would be better to give the junior rank, but a high specialist pay. A field officer who cannot take a parade, who cannot give orders properly, and who perhaps does not always recognise that he is receiving a salute, is a

**THE INSTITUTIONAL LIBRARY—(*continued*).**

mistake. This last criticism is inspired by a Regular officer, and seems sound. The book is excellent.

**The Cause and Prevention of Cancer.** By W. H. Hildebrand. (London: W. H. Smith and Son. 2s. 6d. net. Pp. 163.)

In this little book the author claims "to have been the first to discover that cancer is caused by radium which gets into our bodies principally from drinking water," but the perusal of the evidence brought forward, interesting as are the various points made, fails to show that Dr. Hildebrand can make his claim good. Undeniably one of the most interesting branches of cancer research developed during recent years has been that dealing with the investigation of the relation between morbid growths and radio-activity carried out in the Cancer Department of the Middlesex Hospital under the direction of Dr. Lazarus-Barlow, but it must be noted that those researches do not support this theory. In support of his view the author relies on a number of facts which are capable of alternative explanation. Thus it is suggested that cancerous growths in old people are due to the fact that their tissues in becoming hardened and fibrous hold the deposit of radium more readily than the more elastic tissues of younger people; also that cancer apparently occurs as a result of water-drinking. Again, it is said that the rapid increase in cancer cases during the last century "can only be accounted for by the system of hard-water supplies and water-pipes introduced during that period and by the discontinuance of the use of filters in private houses and elsewhere." Finally, the evidence is unsatisfactory because it is insufficiently supported by statistics. Thus, if it is a fact that "the excess of radium, etc., in old corroded lead water-pipes choked with a scale of mineral deposits and in old corroded zinc cisterns and old boilers in old houses, is the cause of cancer houses," then it should not be difficult to find statistics favouring this statement. The book is interesting because it is an honest attempt to elucidate the relation between radio-activity and morbid cell-proliferation, but the author might well have tended his files and notes carefully for a few more years before hastening into print.

**The Nation's Health.** By Sir Malcolm Morris, K.C.V.O. (London: Cassell and Co., Ltd. 1917. Pp. 152 + x. Price 3s. 6d. net.)

We cannot speak too highly either of the substance or of the method of this book. Its sub-title is "The Stamping Out of Venereal Disease," and no one has more right than Sir Malcolm Morris to speak on such a topic. It was his courageous plea in 1913 which broke down the "conspiracy of silence" and led to the development that has brought venereal disease within the practical grasp of preventive medicine. What can be effected in this direction by knowledge, by education, and by early treatment is a direct outcome of his vigorous intervention. His book speaks to the public, or more particularly to those engaged in local administrative work or in the training and supervision of children and young adults. To write a book on such a subject for such an audience is no easy task. The enterprise demands knowledge, reserve, tact, good taste, and sound judgment. In the present instance all these qualities are readily apparent. The aim is to display in non-technical language what venereal disease is in relation to the individual and to the community, as distinct from the ambition of a medical text-book whose object is instruction in pathology, in diagnosis, and in treatment. Not every medical writer who addresses the public recognises this distinction, and thus we have offered, for general perusal, manuals which are in method works on medicine—less the completeness and accuracy which such a profession would necessarily imply. The timid person who reads books of this order becomes the victim of the horrors at which they hint, while the presumptuous sets himself up as an amateur physician. As might have been confidently anticipated, Sir Malcolm Morris makes no such mistake. He keeps clearly in view the purpose of his pages, and he says what is necessary to this purpose—neither more nor less. His book we regard as a model of what such a book should be, and for it he deserves universal gratitude; by its issue he has added a further contribution to the public services which he has already rendered to the community in connection with a difficult and delicate question. In one of his chapters he discusses certain proposals which have yet to be considered, and while on these he expresses his own view, he is clearly not anxious to force the pace in advance of public opinion. This is quite in harmony with the sound discretion which has characterised throughout the policy of those who are engaged in the new campaign against venereal diseases—active education for public opinion by all means, but not premature compulsory legislation. Towards such an end this book is a substantial and wise contribution.

**A Text-Book of War Nursing.** By Violetta Thurstan. (London: G. P. Putnam's Sons. 3s. 6d.)

This book is primarily intended, as its title shows, for women on active service. It should, however, be read by all nurses, whether they desire to go abroad or not. The book is specially valuable for its picture of the difficulties, emergencies, and accidents likely to occur on active service; and also for the clear directions and excellent practical advice to nurses going abroad for the first time. The author's words about grumbling might well be laid to heart in home hospitals, no less than in Serbia or Italy. She most wisely says that it is much easier to bear real hardships bravely than to be cheerful over cold coffee and damp beds.

The account of the arrangements for transporting the wounded is very interesting, especially, perhaps, the part dealing with the methods on the Eastern Front. Most English nurses have heard something of the work of bringing our own men from France, but the process in the Carpathians and the Caucasus will be quite new to them. Another chapter is devoted to camping and camp hospitals, a subject which is not treated in ordinary nursing manuals. The directions are very clear, and nurses going into camp for the first time could avoid many troubles, large and small, by studying them carefully. Much of the book is necessarily taken up with descriptions of treatment and nursing methods; but these are all discussed from the standpoint of a war hospital, and should therefore be of great value to nurses who are taking up military work for the first time, whether at home or abroad.

**Dainty Dishes for Camp and Home.** By R. Piazzani. (London: John Lane.)

The aim of this book is first and foremost to render the inevitable bully beef more palatable for the soldier. The recipes are, however, a little too elaborate, we fear, for the average mess at the Front, wherein the cook is selected for anything rather than his skill in cooking. They would be admirable for use on a station where some care can be expended on the art of making rather rough and simple fare appetising, and many of the suggestions would prove of great assistance in tempting convalescents to take an interest in their food.

